# Effects of Immersive Virtual Reality on Balance in People With Parkinson’s Disease: A Case Series

**DOI:** 10.7759/cureus.90177

**Published:** 2025-08-15

**Authors:** Lorena Morcillo-Martínez, Natalia Brandín-de la Cruz, Carolina Jiménez-Sánchez, Aitor Garay-Sánchez, Laura Isabel Esteban-Repiso, Lilian Le Roux-Ethève, María Sanz-Mármol, Sandra Calvo

**Affiliations:** 1 Physical Therapy, Faculty of Health Sciences, Universidad San Jorge, Villanueva de Gállego, ESP; 2 Physiotherapy, Miguel Servet University Hospital, Institute for Health Research Aragón (IIS Aragón), Zaragoza, ESP; 3 Physiatry and Nursing, Faculty of Health Sciences, University of Zaragoza, Zaragoza, ESP; 4 Physiotherapy, Institute for Health Research Aragón (IIS Aragón), Zaragoza, ESP

**Keywords:** balance, immersive virtual reality, parkinson's disease, physiotherapy, quality of life

## Abstract

Five people with Parkinson's disease (PD) participated in an immersive virtual reality (IVR) program focused on improving their ability to balance. Over a four-week period, they participated in eight IVR sessions, two per week. Standing balance, functional mobility, gait speed, lower limb strength, incidence of adverse events, quality of life and participant-reported satisfaction were assessed. By comparing pre- and post-intervention outcomes with recognized thresholds of minimal detectable change, improvements in balance, functional mobility, and quality of life were observed. The results of this small case series of five participants suggest that an IVR program intervention seems to have an effect for improving balance, functional mobility, and quality of life in people with PD, warranting further investigation in larger controlled trials.

## Introduction

Parkinson's disease (PD) is considered the second most common neurodegenerative disease in industrialized countries, with a prevalence rate of 150 per 100,000 population [1], affecting approximately 1-2% of the world's population [2], and is one of the 20 leading causes of disability in people over 75 years of age [3]. PD is characterized by a decrease in dopaminergic cells in the basal nuclei, especially in the substantia nigra pars compacta, leading to a loss of dopamine (neurotransmitter responsible for motor control function) and a disruption of connections with other areas of the central nervous system (CNS), such as the premotor and motor areas [1]. This decrease in dopamine leads to a compensatory increase in the activity of other neurotransmitters, notably acetylcholine and glutamate. These neurochemical changes disrupt the excitatory-inhibitory balance within the striatal circuitry, contributing to the pathophysiology of PD [4].

In most cases, PD manifests itself through motor and non-motor signs. The motor signs mainly include resting tremors, hyperresistance, bradykinesia, postural instability, and gait disturbances. The muscles involved in postural control show inadequate regulation, which, combined with increased hyperresistance in the back muscles and limbs, disrupts normal posture and movement patterns, leading to impaired balance and subsequent gait disturbances, which are among the most common disorders in PD [1]. Postural instability is responsible for the increasing number of falls, leading to greater dependence and social isolation, which translates into a reduction in quality of life and health impact, as it affects the ability to work and productivity of people with PD [2].

The main goal of PD treatment is to compensate for the decrease in dopaminergic neurons by administering drugs and, in some cases, deep brain stimulation, a reversible surgical procedure in which the basal nuclei are stimulated by electrodes, is performed. However, the response to these treatments diminishes over time, and they cannot stop the worsening of motor symptoms. For this reason, adjuvant interventions are chosen. In this regard, several studies show the positive effects of usual physiotherapy care in improving motor function through therapeutic exercises for balance, posture, limb activities, and gait, increasing the functionality of the person [5].

Nowadays, recent technological advances have enabled the development of innovative interventions for pathologies of the CNS that complement usual physiotherapy care, such as virtual reality (VR). This technology allows the individual to engage in various tasks in a dynamic virtual environment aimed at improving both the affected structures and the functions associated with the condition. VR-based interventions can be categorized according to the type of devices used, the level of immersion, and the way in which virtual and real information is integrated. Non-immersive VR (NIVR) involves interaction through a screen using an avatar that provides a two-dimensional experience. Semi-immersive VR (SIVR) allows interaction in a virtual environment while maintaining a connection to the real environment, providing a balance between immersion and awareness of reality. Immersive VR (IVR) allows individuals to interact directly with a fully simulated virtual environment and experience realistic scenarios [6].

Several studies have reported positive results using NIVR in people with PD, leading to significant improvements in balance [7-9] and gait [10]. On the other hand, IVR has been shown to improve upper limb motor skills and oculomotor coordination [11] and increase gait efficiency by increasing the stride length [12] in PD. In addition, the combination of treadmill training and IVR improves both balance and gait in people with PD [13]. These therapeutic effects are likely because IVR provides external feedback that compensates for disrupted neural pathways, thereby facilitating motor learning and cognitive processing to support functional recovery. Neuroimaging studies suggest that IVR increases activity in brain regions rich in mirror neurons, particularly in the frontal and parietal lobes, which underlie motor execution and sensory integration, respectively [6].

Despite promising, albeit sparse, evidence for IVR in Parkinson’s rehabilitation, there are no studies specifically targeting its impact on dual-tasking and static and dynamic postural control in this population [11]. Therefore, the aim of this case series is to evaluate the effects of IVR in improving balance as a primary outcome, while also evaluating its impact on gait speed, functional mobility, lower limb strength, and quality of life as secondary outcomes in people with PD. In addition, it aims to determine the incidence of adverse symptoms associated with IVR treatment and assess patient satisfaction with the intervention.

## Case presentation

Study design

This study is a pre-post case report of five PD participants assessed between February and May 2025, after signing an informed consent form on the first day of enrollment. Participants were recruited by the Aragon Association of Parkinson (Zaragoza, Spain) and the intervention was approved by the Ethics Committee of Aragon (reference number PI24/501).

Eligibility Criteria

The inclusion criteria were as follows: (i) Diagnosis of PD confirmed by a neurologist; (ii) Aged 55 to 90 years; (iii) Hoehn & Yahr Scale score of 1 to 4; (iv) Cognitive level of 24 or higher on the Mini Mental Examination Test; and (v) Ability to stand upright without assistance. The exclusion criteria were: (i) Visual or auditory impairments that could interfere with the perception of information or trigger dizziness or epilepsy; (ii) Musculoskeletal conditions that hinder or prevent the execution of exercises or the ability to maintain a standing position for at least 20 minutes; and (iii) Psychiatric disorders such as moderate or severe depression diagnosed in the last year and impulse control disorders diagnosed in the last year. The resignation criteria were: (i) Occurrence of severe adverse effects while using the IVR (score of more than 20 points on the SSQ scale); (ii) Adherence rate of less than 90% to the intervention; and (iii) refusal to continue.

Case presentation

Case 1

A 69-year-old woman was diagnosed with PD by a neurologist six years ago. She had an intermediate level of education. Her balance had been impaired since 2022 (especially balance on the left leg). She had no history of PD or other Parkinsonian disorders in her family. In 2000, she underwent surgery on the meniscus of her right knee. Her past medical history was unremarkable. Her pharmacological management included consistent carbidopa/levodopa doses. Her cognitive abilities were adequate, and her functional impairment was 1 on the Hoehn & Yahr scale.

Case 2

A 63-year-old woman was diagnosed with PD in 2017. Her educational level was intermediate. She has no other pathology; however, there are cases of PD in her family. Her pharmacological management included consistent carbidopa/levodopa, pramipexole, amantadine, opicapone, safinamide, omeprazole, and clorazepate dipotassium doses. She has no cognitive impairment, and her functional impairment was 2 on the Hoehn & Yahr scale.

Case 3

A 68-year-old man was diagnosed in 2019 with PD. He obtained a higher level of education. He has had surgery for a hernia and has other conditions such as high cholesterol and arterial hypertension. In his family, his maternal aunts and paternal grandfather have PD. His pharmacological management included consistent carbidopa/levodopa, amantadine, opicapone, safinamide, and atorvastatin doses. He had an adequate cognitive level, and his functional impairment was 1 on the Hoehn & Yahr scale.

Case 4

A 78-year-old man who was diagnosed with PD four years ago. He had an intermediate level of education. He had undergone prostate surgery and narrow lumbar canal surgery. He had no history of PD or other Parkinsonian disorders in his family. He received concomitant medications including levodopa/carbidopa, rasagiline, opicapone, candesartan/hydrochlorothiazide, acetylsalicylic acid, atorvastatin, and quetiapine. His cognitive abilities were fine, and his functional impairment was 2 on the Hoehn & Yahr scale.

Case 5

A 75-year-old man was diagnosed with PD in 2022. He had an intermediate level of education. His medical history included benign prostatic hyperplasia and Meniere's syndrome; neither of these conditions currently affected him. His father also had PD. He received concomitant medications including levodopa/carbidopa, opicapone and quetiapine. As for his cognitive abilities, he had no problems, and his functional impairment was 2 on the Hoehn & Yahr scale.

The sociodemographic and clinical characteristics of the five cases are presented in Table 1.

**Table 1:** Sociodemographic and Clinical Characteristics.

Case	Age	Gender	Weight (Kg)	Height (cm)	Education Level	Employment Status	Year of Diagnosis	Disease Degree (Hoen&Yahr Scale)	Medications
Case 1	69	Female	78	168	Secondary	Retiree	2019	1	Levodopa/carbidopa.
Case 2	63	Female	75	150	Secondary	Retiree	2017	2	Levodopa/carbidopa. Pramipexole. Amantadine. Opicapone. Safinamide. Omeprazole. Clorazepate dipotassium.
Case 3	68	Male	100	175	University	Retiree	2019	1	Levodopa/carbidopa. Amantadine. Opicapone. Safinamide. Atorvastatin.
Case 4	78	Male	81	171	Secondary	Retiree	2021	2	Levodopa/carbidopa. Rasagiline. Opicapone. Candesartan/hydrochlorothiazide. Acetylsalicylic acid. Quetiapine.
Case 5	75	Male	90	160	Secondary	Retiree	2022	2	Levodopa/carbidopa. Opicapone. Quetiapine.

Intervention

After recruitment, the physiotherapist in charge of the assessment visited the Aragon Association of Parkinson's to carry out the assessments. In the initial phase, participants completed the written scales and followed the physical examination. After completing the initial assessments, the IVR intervention began in the following session, which consisted of eight sessions. Upon completion of the IVR intervention, the physiotherapist who conducted the initial assessment repeated the assessment.

The intervention consisted of the application of eight sessions, two sessions per week for four weeks to treat balance, gait speed, functional mobility and strength of the lower limbs through a rehabilitation program divided into two sections. Each session lasted 40 minutes and was divided into two 20-minute sections (Table 2).

**Table 2:** Intervention.

Intervention	
Section 1 (20 minutes)	Section 2 (20 minutes)
15 repetitions of the eccentric exercise of the anti-gravity muscles: Trunk, Knee flexors, Knee extensors, and Hip extensors	Balance exercises (static and dynamic) using the IVR: Five scenarios were used to work static or dynamic balance with specific stability tasks. Dual task performance: maintaining balance and solving motor and/or cognitive tasks. Four difficulty levels (from basic to advanced).

The first part consisted of a usual physiotherapy intervention in which a series of 15 repetitions of the eccentric exercises were performed for the antigravity muscles, such as the trunk, knee flexors and extensors, and hip extensors. These exercises were performed in a sitting or standing position, depending on which muscle was being worked, with a slow contraction speed to avoid compensation. The duration of the eccentric contraction of each exercise was adapted to each participant, with a maximum of 3 seconds of eccentric contraction [14].

**Figure 1:**
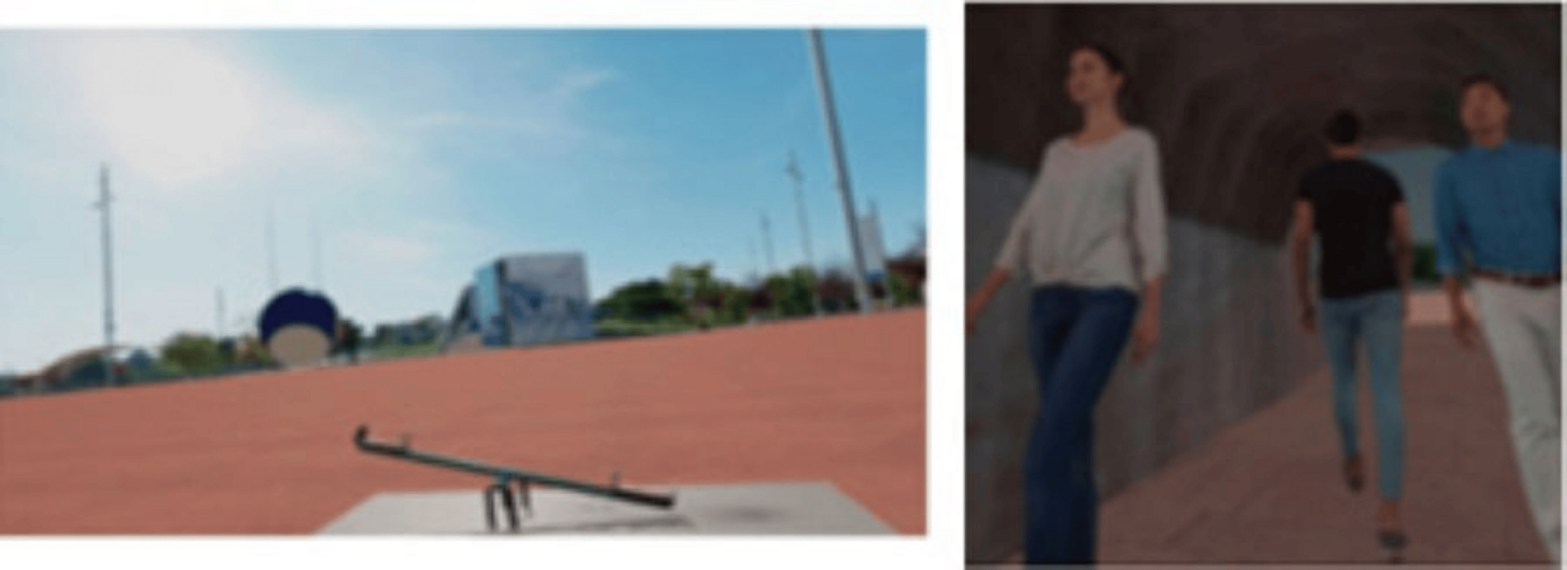
Scenarios of IVR. IVR: Immersive virtual reality

The second part consisted of balance exercises using the IVR Oculus Quest 2 and the FISIOVR software (Abaco Digital, Zaragoza, Spain). The program consisted of an IVR with a series of spherical images of urban frames familiar to participants, allowing them to experience situations of static and dynamic balance while performing different postural activities in a realistic, simulated 360º reality that allows the person to move and explore the environment with greater freedom of movement. The IVR intervention program consisted of four levels of difficulty, from basic level 1 to advanced level 4, generally varying the width and stable/unstable support surface. The level of difficulty was individualized to each participant's ability to ensure a tailored experience and to facilitate progression to more challenging exercises. Progression occurred when participants successfully completed an exercise with a maximum imbalance of 1. A total of five scenarios (Figure 1) were used, which were the same in all sessions and in which different types of balance (static or dynamic) and specific stability tasks (quiet, reactive or proactive) were performed. The participants had to perform a dual task of maintaining balance and solving motor and/or cognitive tasks using auditory, visual and/or internal mental inputs (e.g. mental counting, internal numerical calculations, working memory, such as counting the number of balls, indicating how many people are in the tunnel, how those people are dressed etc.).

Participants had not previously received IVR treatment. For this reason, at the beginning of the first RV session and for initial adaptation to the VR glasses, participants were seated comfortably, given VR glasses, and the frames and lenses were adjusted to ensure a clear and comfortable vision. They then identified objects and landmarks to confirm visual accuracy. During this initial contact and familiarization with VR, subjective comments on distortion, discomfort or disorientation were collected to assess the initial adaptation.

Outcomes

Primary Outcome

The Mini-Balance Systems Test (Mini-BESTest) was used to measure balance impairment. The Mini-BESTest assesses static and dynamic balance disorders and determines the risk of falling by evaluating 14 items divided into four categories: anticipatory, reactive postural control, sensory orientation and dynamic gait. The dynamic gait also includes a Time Up & Go (TUG) and a Time Up & Go Dual Task TUG-DT). These items are scored from 0 to 2 points, with 0 indicating the lowest and 2 the highest level of function, with a maximum score of 28 (no risk of falling). The Mini-BESTest has been shown to be reliable in people with PD, and it has excellent test-retest reliability (ICC=0.92) [15,16].

Secondary Outcomes

The 10-Meter Walk Test (10MWT) was used to assess the participants' gait speed. The participant was asked to walk 10 meters on a flat surface. Three repetitions were performed at the participant's comfortable walking speed followed by three repetitions at a fast-walking speed. The average of the three repetitions of each gait in meters/second is determined. This test has excellent test-retest reliability at a comfortable pace in individuals with PD (ICC=0.81) [17].

The 30 Second Sit to Stant Test (30CST) is part of the Senior Fitness Test (SFT). It was used to evaluate lower limb strength. This test had already been used as a stand-alone test for other pathologies. The participant was seated in the chair, keeping his back straight, with his feet on the floor slightly behind his knees and his arms folded across his chest. He had to perform as many squats as possible in 30 seconds. This test has excellent test-retest reliability in community-dwelling elderly (ICC=0.95) [18].

The Time Up & Go (TUG) test assesses the participant's functional mobility during walking, as well as the risk of falling. The participant should be seated in a chair with armrests and, after being instructed to "go," stand up, walk in a straight line for three meters, turn around, and sit back down in the chair. This test has high intratester reliability in people with PD (ICC=0.98) [19].

The Time Up & Go Dual Task (TUG-DT) test was used to measure the participant's functional mobility during walking, as well as the risk of falling while performing a dual task. The participant should be seated in a chair with armrests and, after being instructed to "go," stand up, walk in a straight line for three meters, turn around, at the same time the person is counting down aloud from the number 70, subtracting 3 each time and sit back down in the chair [20].

The SSQ (Simulator Sickness Questionnaire) was used to assess the occurrence of adverse symptoms during the use of the IVR intervention. It was also used as a criterion for withdrawal from the IVR program: if the participant reached a score of 20, he was asked to leave the study due to the severe symptoms indicated in the scale. This questionnaire consists of 16 items divided into three sections: Ocular Motor Function, Nausea, and Disorientation. The researcher should record the presence and intensity of each item as it occurs. Scores below 5 are considered insignificant symptoms, 5-10 as minimal, 10-15 as significant, 15 to 20 as worrying and more than 20 as severe symptoms [21]. 

PDQ-39 (Parkinson's Disease Questionnaire-39), which is a self-administered questionnaire, consists of 39 items, divided into eight dimensions, that assess health-related quality of life over the past month in people with PD. The PDQ-39 questionnaire has been shown to be reliable in people with PD, with high test-retest reliability (ICC=0.68-0.95) [22].

Satisfaction with treatment: Participants' satisfaction was measured using an ad hoc questionnaire on a 5-point Likert scale, with 5 being very satisfied and 1 being very dissatisfied.

Results

All participants completed all five scenarios during the eight sessions of the IVR program. Each participant achieved difficulty levels 3 and 4 in each scenario.

Balance Impairment

All participants improved on balance as measured by the Mini-BESTest, with case 3 showing the greatest improvement. Overall, all participants showed an increase in the score of 6 points (31.57%) from M0 19 ± 2.23 to M1 25 ± 1.64 (Table 2) exceeding the Minimum Detectable Change (MDC) of 17.1% established for people with PD [15]. Changes were observed in several test domains, including anticipatory adjustments (preparatory postural responses), reactive postural control (responses to external perturbations), sensory orientation and dynamic gait, with the improvement in anticipatory adjustments and dynamic gait being the most significant, with increases of 1.2 and 3.6 points, respectively (Table 3).

**Table 3:** Balance Impairment. Mini-BESTest: Mini Balance Evaluation Systems Test; n: points; M0: Pre-intervention; M1: Post-intervention; CS: Change Score; SD: Standard Deviation.

Variable	Case 1			Case 2			Case 3			Case 4			Case 5			Mean ± SD		
	M0	M1	CS	M0	M1	CS	M0	M1	CS	M0	M1	CS	M0	M1	CS	M0	M1	CS
MiniBESTest (n/28)	22	27	5 (17.85%)	16	23	7 (25%)	18	26	8 (28.57%)	20	24	4 (14.29%)	19	26	7 (25%)	19 ± 2.23	25 ± 1.64	6 ± 0.59 (31.57%)
Anticipatory (n/6)	4	5	1	2	3	1	3	5	2	4	5	1	4	5	1	3.4 ± 0.89	4.6 ± 0.89	1.2 ± 0.89 (35.29%)
Reactive postural control (n/6)	5	6	1	4	6	2	5	6	1	5	5	0	5	6	1	4.8 ± 0.44	5.8 ± 0.44	1 ± 0.44 (20.83%)
Sensory orientation (n/6)	5	6	1	4	6	2	6	6	0	6	6	0	6	6	0	5.4 ± 0.89	6 ± 0	0.6 ± 0.89 (11.11%)
Dynamic Gait (n/10)	8	10	2	6	8	2	4	9	3	4	8	4	5	9	5	5.2 ± 1.78	8.8 ± 0.83	3.6 ± 0.95 (69.23%)

Gait Speed, Limb Strength and Functional Mobility

Regarding secondary variables (Table 4), slight improvements in gait speed measured with the 10MWT were generally observed in all participants, with an increase of 0.186 ± 0.2333 m/s in comfortable gait speed and 0.248 ± 0.2096 m/s in fast gait speed, exceeding the MDC thresholds of 0,18 m/s and approaching the MDC thresholds 0.25 m/s, respectively [23]. In comfortable walking speed only case 5 exceeded the MCD. At fast walking speed, cases 2 and 3 exceeded the MCD.

**Table 4:** Gait Speed, Limb strength, and Functional Mobility. 10MWT: 10 Meter Walk Test; 30CST: 30 Second Sit to Stand Test; TUG: Time Up and Go test; m/s: meters per second; M0: Pre-intervention; M1: Post-intervention; SD: Standard Deviation.

Variable		Participant	Assessment		Change Score
			M0	M1	
10 MWT (m/s)	Comfortable walking speed	Case 1	2.15	2.32	0.17
		Case 2	2.47	2.43	-0.04
		Case 3	2.24	2.15	0.09
		Case 4	1.45	1.59	0.13
		Case 5	2.28	2.86	0.58
		Mean ± SD	2.118 ± 0.3912	2.27 ± 0.4617	0.186 ± 0.2333
	Fast walking speed	Case 1	3.31	3.42	0.11
		Case 2	2.64	3	0.36
		Case 3	2.99	3.04	0.05
		Case 4	1.96	2.52	0.56
		Case 5	3.38	2.54	0.16
		Mean ± SD	2.856 ± 0.5805	2.904 ± 0.3787	0.248 ± 0.2096
TUG (s)	Normal	Case 1	15.86	12.13	3.73
		Case 2	15.76	8.6	7.16
		Case 3	12.01	8.08	3.93
		Case 4	15.93	9.99	5.94
		Case 5	11.57	8.41	3.16
		Mean ± SD	14.22 ± 2.23	9.442 ± 1.66	4.784 ± 1.69
	Dual Task	Case 1	21.71	12.41	9.3
		Case 2	29.83	12.32	9.49
		Case 3	17.5	10.85	6.65
		Case 4	23.37	15.65	7.72
		Case 5	14.74	11.37	3.37
		Mean ± SD	21.43 ± 5.80	12.52 ± 1.86	7.306 ± 2.49
30CST		Case 1	11	14	3
		Case 2	12	17	5
		Case 3	9	11	2
		Case 4	8	12	4
		Case 5	20	20	0
		Mean ± SD	12 ± 4.74	14.8 ± 3.70	2.8 ± 1.92

Changes were noted in functional mobility as measured by the TUG and TUG-DT, a decrease of 4.784 ± 1.69 seconds and 7.306 ± 2.49 seconds, respectively. These values were above the established MDC of 3.05 seconds for TUG [24] and 2.52 seconds for TUG-DT [25], indicating a relevant change.

Performance on the 30CST, used to assess lower limb strength, also increased by 2.8 ± 1.92 repetitions, which is below the MDC of 3.3 repetitions [26]. Only cases 2 and 4 achieved an improvement of 5 and 4 repetitions respectively, above the MDC.

Quality of Life

The PDQ-39 scale showed relevant changes in quality of life (Table 5), with a decrease in score of 11 points between the measurements the pre-intervention and after the IVR sessions, exceeding the MDC of 4.72 points [27] The greatest change was seen in the mobility, which decreased from 14 ± 7.64 points pre-intervention to 5.6 ± 3.84 points post-intervention.

**Table 5:** Quality of Life Assessment using PDQ-39. PDQ-39: Parkinson’s Disease Questionaire-39; n: points; CS: Change Score; M0: Pre-intervention; M1: Post-intervention; SD: Standard Deviation.

Variable	Case 1			Case 2			Case 3			Case 4			Case 5			Mean ± SD		Change Score
	M0	M1	CS	M0	M1	CS	M0	M1	CS	M0	M1	CS	M0	M1	CS	M0	M1	M0-M1
PDQ-39- (n/100)	55	39	16	69	48	20	32	17	15	42	26	16	9	21	-12	41.4 ±22.83	30.4 ± 13.29	11±9.54
Mobility	19	13	6	22	11	11	8	1	7	17	7	10	4	3	1	14 ± 7.64	5.6 ± 3.84	8.4±3.8
ADLs	6	4	2	8	7	0	6	5	1	4	3	1	5	5	0	5.8 ± 1.48	4.8 ± 1.48	1±1.48
Emotional Well-Being	10	7	3	14	7	7	8	4	4	5	3	2	0	0	0	7.4 ± 5.27	4.2 ± 2.94	3.2±2.33
Stigma	0	1	-1	1	1	0	0	0	0	0	0	0	0	12	-12	0.2 ± 0.44	2.8 ± 5.16	-2.6±4.72
Social Support	0	0	0	1	0	1	1	0	1	0	0	0	0	0	0	0.4 ± 0.54	0 ± 0	0.4±0.54
Cognition	8	6	2	10	9	1	6	5	1	6	5	1	0	1	-1	6 ± 3.74	5.2 ± 2.86	0.8±0.88
Communication	5	2	3	7	7	0	2	1	1	3	4	-1	0	0	0	3.4 ± 2.70	2.8 ± 2.77	0.6±0.07
Bodily Discomfort (PDSI)	7	6	1	6	6	0	1	1	0	7	4	3	0	0	0	4.2 ± 3.42	3.4 ± 2.79	0.8±0.63

Satisfaction With Treatment and Adverse Symptoms

The participants' satisfaction with the intervention was rated as very high, 4.8 points on a 5-point Likert scale.

Regarding the potential occurrence of adverse effects, no participants reported any unfavorable symptoms during the IVR sessions. Accordingly, all participants obtained a score of 0 on the SSQ, indicating an absence of cybersickness or related discomfort.

## Discussion

This series of cases shows that IVR treatment may lead to an improvement in both static and dynamic balance, while also improving functional mobility and quality of life. The IVR intervention was well tolerated, with no adverse effects reported, and the participants expressed high satisfaction with the treatment.

As for the main variable, balance, an improvement was observed, exceeding the MCD of 17.1% [15]. These results are consistent with previous research, IVR combined with cognitive‑motor dual‑task training has been shown to improve balance as measured by the Berg Balance Scale (BBS) [27], and a 12 session IVR program combined with antigravity treadmill training (three 30 minute sessions per week) resulted in significant improvements on balance on the Tinetti Scale in people with PD [28]. On the other hand, several studies have reported positive results from the use of NIRV in people with PD, such as the study by Esculier et al. [7], which showed improvements in balance with an increase of 4 out of 28 points on the Tinetti scale after 18 sessions of 30 minutes. However, we cannot compare our results since the scale we used is different. Other authors, such as Kim et al., indicate improvement in balance and gait by showing significant improvements in Mini-BESTest scores after a single 20-minute gait session in a virtual non-immersive environment, supporting the potential of NIVR as a complementary strategy in balance rehabilitation [29].

Regarding functional mobility assessed by the TUG and TUG-DT, a clinical improvement was observed exceeding the MCD threshold of 3.05 seconds [26]. This result contrasts with the study by Honzíková et al., in which no significant changes in TUG-DT performance were observed after IVR treatment. This discrepancy could be due to differences in intervention design. In our study, all IVR scenarios included cognitive tasks that potentially improved dual-task performance, whereas in the aforementioned study, such tasks were not consistently included. Furthermore, differences in the dosage of the intervention could also explain the divergent results. Our participants completed eight 40-minute sessions (including 20 minutes of IVR per session), while participants in the Honzíková study received only one 30-minute session of usual physiotherapy per week and one or two IVR sessions of 10-20 minutes [28]. A study reported improvements in TUG and TUG-DT performance after 12 weeks of IVR-based training, although these changes were not significant, highlighting the complexity of dual-task rehabilitation outcomes [30].

No clinical improvement in gait speed was observed, a post-intervention although in comfortable walking speed, participant 5 exceeded the MCD, and in fast walking speed, participants 2 and 3 also exceeded it. This could be attributed to the fact that the exercises specifically targeted static and dynamic balance, and not at walking speed. These results are consistent with a systematic review of PD, which concluded that virtual reality-based rehabilitation had no significant effect on gait speed [31].

Similarly, no clinical changes were found in lower limb strength measured with 30CST, since only 2 of the 5 participants showed improvement, being above the MDC. This may be explained by the frequency of the intervention, as only two sessions per week were conducted. Previous research suggests that at least three weekly sessions are generally required to achieve measurable improvements in muscle strength [32].

In terms of quality of life, four of our participants reported a significant increase, surpassing the MCD of 4.72 points [27]. These results are consistent with those obtained by Pedreira et al., who showed improvement in the quality of life in persons with PD who had used NIVR compared to the group that received usual physiotherapy intervention [33].

Regarding participants' satisfaction with the intervention, it was rated at 4.8 points on a 5-point Likert scale was obtained, confirming other authors' data suggesting that IVR is associated with greater participant engagement, sustained motivation, and potential cognitive benefits, which may improve adherence to rehabilitation programs over time [34]. The results of this case series after eight sessions of IVR-based intervention support this perspective, as evidenced by the positive outcomes and high participant satisfaction with treatment.

Consistent with our findings and previous research, IVR may contribute to the recovery of impaired function in neurological populations by improving motor learning and cognitive processing. This is achieved in part through enhanced external feedback, which may help compensate for damaged neural networks. Neuroimaging studies have reported increased neuronal activity following IVR sessions, particularly in brain regions associated with mirror neuron systems, such as the frontal and parietal lobes, which play a key role in motor control, sensory integration, and language processing [6].

However, this study has several limitations. In this case series, the sample size is small and there is no control group, so the results cannot be generalized to the general PD population. Larger studies with robust sample sizes and group controls are needed to assess the efficacy and reproducibility of IVR interventions on balance rehabilitation for people with PD. Furthermore, in the absence of long-term follow-up data, conclusions about the durability of the observed improvements are limited. Future research should include evaluations of longer time periods, such as three months post-intervention, to assess the sustainability of therapeutic effects.

## Conclusions

Based on the results obtained with our five participants, it can be deduced that the IVR rehabilitation program seems to have an effect on improving static and dynamic balance, increasing functional mobility, and improving quality of life in people with PD. This exploratory case series highlights the need for future research with larger samples to determine whether the positive effects obtained on static and dynamic balance after the application of IVR are maintained over time.
